# A New Approach for Chagas Disease Screening Using Serum Infrared Spectroscopy and Machine Learning Algorithms

**DOI:** 10.1021/acsinfecdis.5c00377

**Published:** 2025-08-28

**Authors:** Matthews Martins, Ângelo Antônio Oliveira Silva, Felipe Silva Santos de Jesus, Emily Ferreira Santos, Daniel Dias Sampaio, Wanderson Romão, Fred Luciano Neves Santos, Valerio G. Barauna

**Affiliations:** † Department of Physiological Sciences, 28126 Federal University of Espírito Santo, Av. Mal. Campos, 1468–Maruípe, Vitória, Espírito Santo 29047-105, Brazil; ‡ Advanced Health Public Laboratory, Goncalo Moniz Institute, Oswaldo Cruz Foundation, Salvador, Bahia 40296-710, Brazil; § Interdisciplinary Research Group in Biotechnology and Epidemiology of Infectious Diseases (GRUPIBE), Goncalo Moniz Institute, Oswaldo Cruz Foundation, Salvador, Bahia 40296-710, Brazil; ∥ Federal Institute of Espírito Santo (IFES), Av. Min. Salgado Filho, 1000–Soteco, Vila Velha, Espírito Santo 29106-010, Brazil; ⊥ Integrated Translational Program in Chagas disease from Fiocruz (Fio-Chagas), Oswaldo Cruz Foundation, Rio de Janeiro, Rio de Janeiro 21040-900, Brazil

**Keywords:** chagas disease, spectroscopy, machine learning, screening, serum

## Abstract

Chagas disease (CD) affects an estimated 6–7 million people worldwide, predominantly in Latin America. However, migration has expanded its geographic reach. Diagnosing chronic CD is challenging due to low parasitemia and the limitations of existing serological assays. This study evaluates the diagnostic potential of attenuated total reflectance Fourier-transform infrared (ATR-FTIR) spectroscopy combined with machine learning (ML). A total of 100 serum samples (49 CD-positive, 51 negative controls) were analyzed using ATR-FTIR spectroscopy under two conditions: (i) dry analysis (air-dried samples) and (ii) wet analysis (direct serum analysis). Spectral data were processed using ML algorithms, including logistic regression (LR), partial least-squares discriminant analysis (PLS-DA), random forest (RF), and extreme gradient boosting (XGBoost) for sample classification. The best-performing models were LR for dry data set (accuracy and F1-score: 93%) and XGBoost for the wet data set (accuracy and F1-score: 87%). The area under the receiver operating characteristic (ROC) curve (AUC) was 0.99 and 0.92 for the dry and wet data sets, respectively. The robustness and reliability of the model were confirmed through permutation tests. These results demonstrate that ATR-FTIR spectroscopy combined with ML is a promising diagnostic tool for CD. Despite the study’s limited sample size, results suggest this approach could serve as a cost-effective alternative to conventional serological assays, particularly in resource- constrained settings. Further validation with larger data sets and diverse control groups is essential to assess its specificity and clinical applicability. If successful, this method could significantly enhance early diagnosis and improve disease managements strategies for CD.

## Graphical abstract

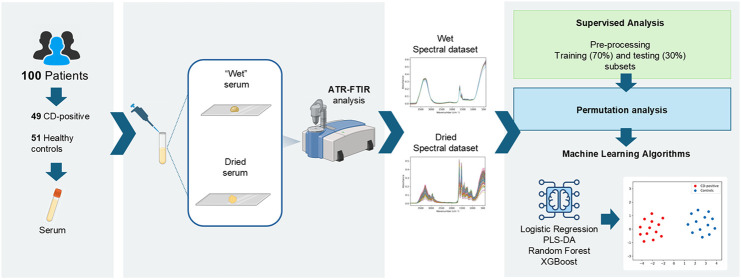

Chagas disease (CD), caused by the protozoan *Trypanosoma cruzi*, is a neglected tropical disease that remains a significant global public health challenge. An estimated 6–7 million people are affected worldwide, with the highest burden concentrated in 21 endemic Latin American countries.^1^ However, increasing global migration has facilitated the spread of CD to nonendemic regions, including the United States, Canada, Europe, and parts of Africa, the Eastern Mediterranean, and the Western Pacific, transforming it into an emerging global health concern.^2,3^ The primary mode of *T. cruzi* transmission is vectorial, occurring through contact with the feces and urine of infected triatomine insects (“kissing bugs”) during or after a blood meal. Other transmission routes include ingestion of contaminated food or beverages, congenital transmission, blood transfusions, organ transplantation, and, less frequently, laboratory accidents.^4^

CD progresses through acute and chronic phases. The acute phase is often asymptomatic or presents with mild, nonspecific symptoms, making early detection challenging. If untreated, the infection advances to the chronic phase, where approximately 60–70% of individuals remain asymptomatic (indeterminate phase), with no detectable abnormalities on electrocardiography (ECG), chest radiography, or gastroenterological contrast imaging. However, 30–40% develop severe, potentially life-threatening complications, primarily affecting the cardiovascular and digestive systems.^5^ Cardiac manifestations include progressive heart failure, arrhythmia, and cardiomegaly, while digestive complications may involve esophageal (megaesophagus) and colonic (megacolon) enlargement.^5,6^ These conditions significantly impair patients’ well-being and health-related quality of life and contribute to increased morbidity and mortality.^7^

Diagnosing chronic CD is particularly challenging due to low and intermittent parasitemia. As a result, laboratory diagnosis relies on indirect serological methods, including enzyme-linked immunosorbent assay (ELISA), immunofluorescence (IIF), hemagglutination (IHA), and chemiluminescence (CLIA).^8^ However, discrepancies in test performance are frequently reported,^9–11^ necessitating the use of two independent serological methods for diagnostic confirmation, as recommended by the World Health Organization (WHO).

The demand for more reliable screening and diagnostic tools has driven advancements in healthcare technologies. Artificial intelligence (AI) and machine learning (ML) have shown great promise in predictive modeling, image processing, and risk assessment.^12,13^ These algorithms can autonomously analyze large and complex data sets, identify patterns, and enhance diagnostic precision, thereby reducing uncertainty and improving medical decision-making.^14^

This study proposes a rapid, reagent-free, and pathogen-agnostic diagnostic approach using attenuated total reflectance Fourier-transform infrared (ATR-FTIR) spectroscopy, a well-established technique for analyzing biological materials. ATR-FTIR spectroscopy captures interactions between infrared light and biofluid samples, generating spectral data that reflect their biochemical composition.^15^ ML algorithms are then applied to process this spectral information, reducing dimensionality and constructing predictive models to classify individuals based on the probability of *T. cruzi* infection.^16^ This proof-of-concept study aims to evaluate the feasibility of ATR-FTIR spectroscopy combined with ML for the diagnosis of chronic CD, addressing critical gaps in current CD diagnosis and management.

## Results

The raw spectra collected for both data sets are presented in Figure 1. Panel A displays the spectra of wet samples, while panel C shows those of dried samples. In this figure, the prominent interference of water OH bonds on the spectral characteristics is clearly observed. As shown in Figure 1A, water exhibits strong absorption in the IR region, dominated by the −OH stretching mode at 3500 cm^–1^ and the −OH bending mode at 1640 cm^–1^. The dry-film measurement (Figure 1C) eliminates the spectral contribution of water, thereby enhancing the biomolecular absorbance signals. The mean spectra of positive and negative samples for both data sets are illustrated in panels B (wet samples) and D (dried samples).

**Figure 1:**
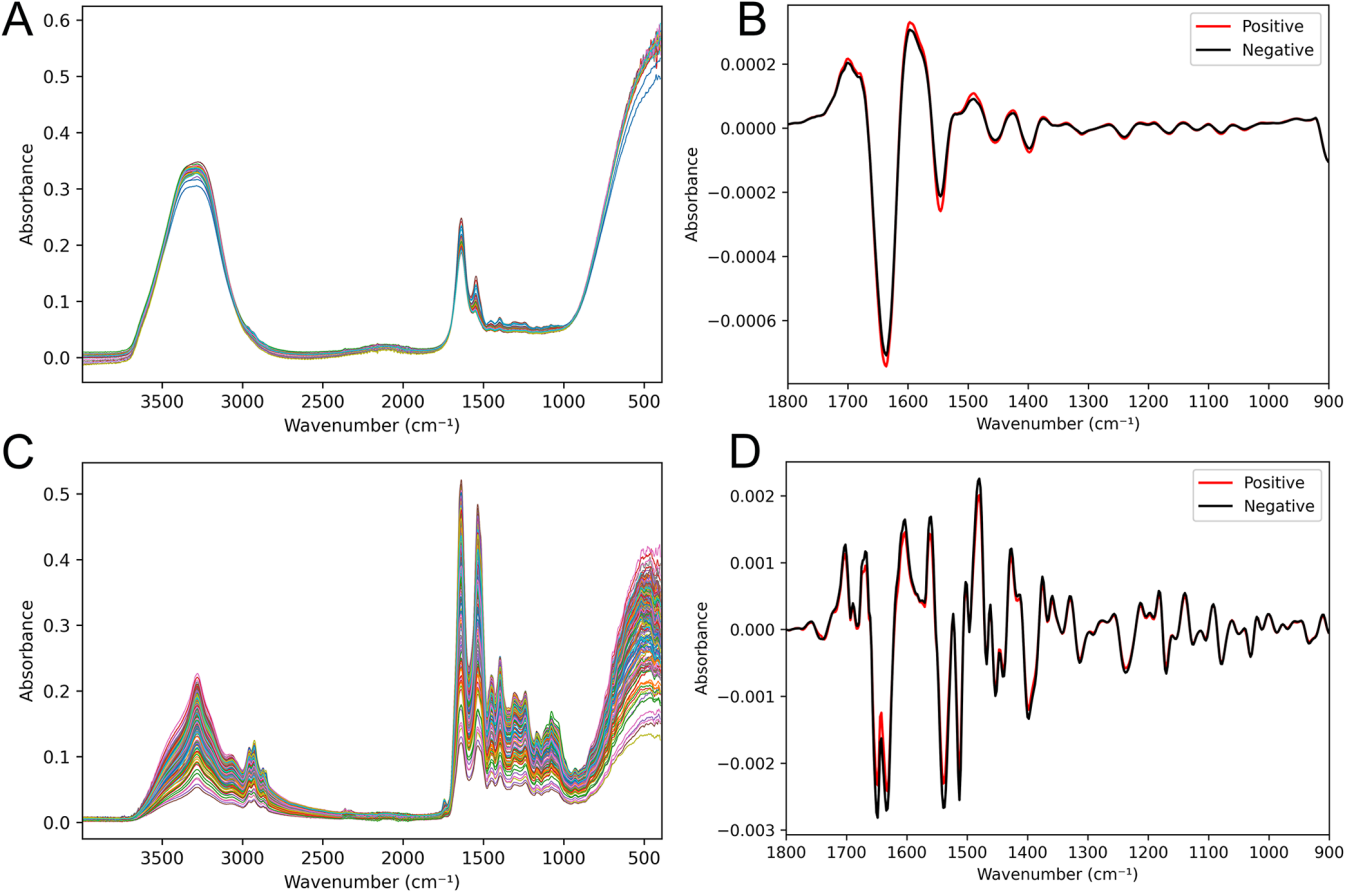
Raw spectra of the wet data set (A). The mean of the wet data set’s positive and negative preprocessed spectra (B). Raw spectra of the dry data set (C). The mean of the dry data set’s positive and negative preprocessed spectra (D).

In both cases, the most significant spectral differences between groups are observed in the 1500–1700 cm^–1^ region, corresponding to key biochemical variations associated with disease status. In blood serum, this spectral region contains bands associated with peptide vibrations of proteins,^15^ including Amide II (N–H bending and C–N stretching modes) between 1500 and 1600 cm^–1^ and Amide I (C=O peptide vibration and C=O stretching) between 1600 and 1700 cm^–1^.

The classification performance of the ML models for both data sets is summarized in Table 1. Performance metrics were evaluated based on the models’ ability to correctly classify the test set, which consisted of unseen samples. In the dry data set, logistic regression was the most effective classifier, achieving an accuracy and F1-score of 93%. In the wet data set, XGBoost exhibited the best performance, with an accuracy and F1-score of 87%.

**Table 1:** Performance Metrics for the Test Set for Both Dry and Wet Datasets

	performance metrics			
model	sensitivity	specificity	accuracy	F1-score
Dry Data Set				
logistic regression (LR)	93%	93%	93%	93%
PLS-DA	87%	93%	90%	90%
Wet Data Set				
random forest (RF)	80%	87%	83%	83%
XGBoost	87%	87%	87%	87%

The AUC value is a key metric for assessing the clinical applicability of classifiers. Figure 2 presents the ROC curves for the four classification models. LR achieved the highest performance in the dry data set, while XGBoost demonstrated the best results in the wet data set. Although the overall performance metrics were higher in the dry data set, the wet data set also exhibited excellent predictive capability. Considering its rapid analysis time, ATR-FTIR spectroscopy using wet samples represents a promising approach for CD diagnosis.

**Figure 2:**
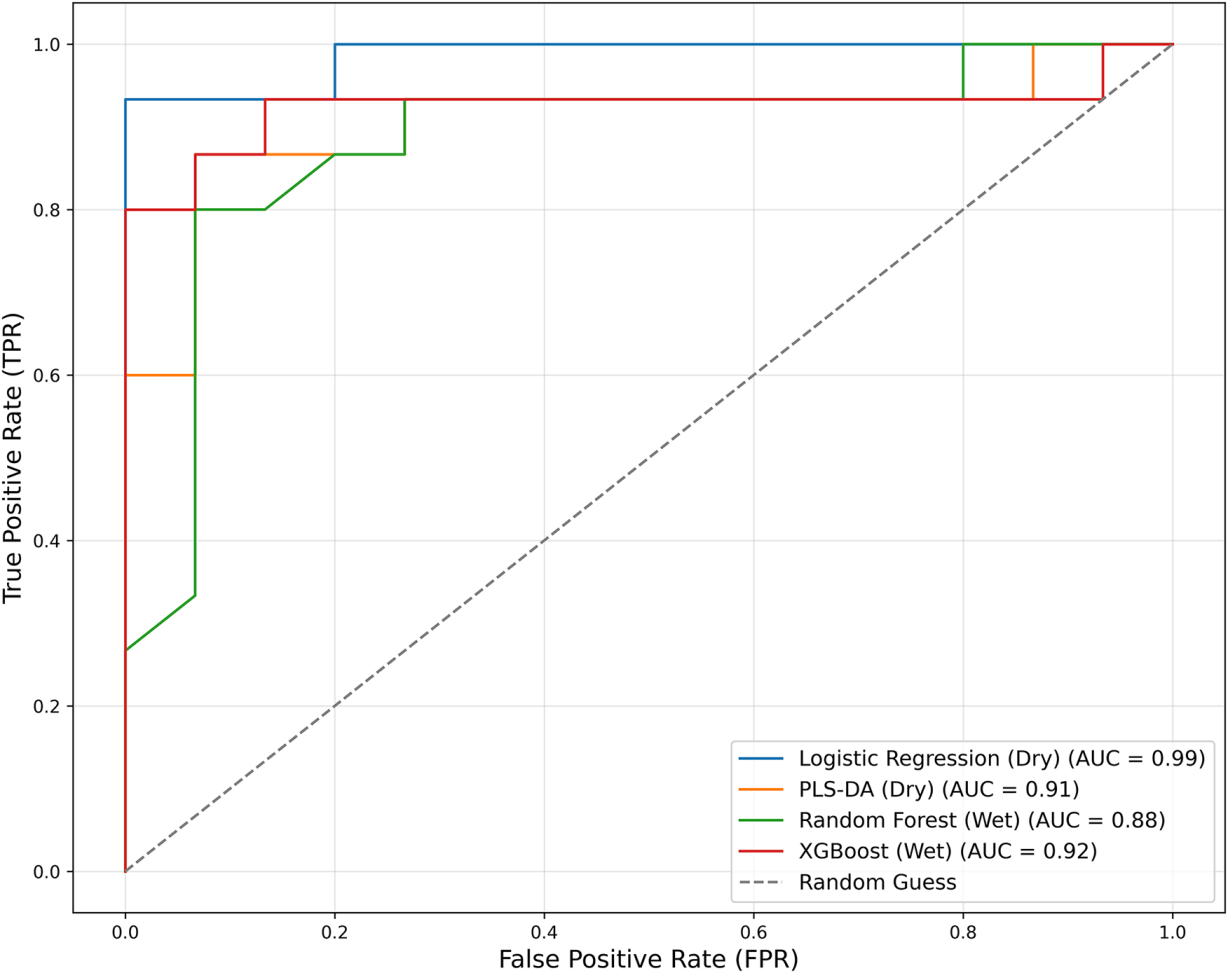
ROC curve of the classification models constructed for dry and wet data sets.

Permutation tests were performed to validate the robustness of the models. The results for the dry data set (Figure 3A,B) and wet data set (Figure 3C,D) demonstrate that the real models, constructed using the original class vector (dashed red line), fall outside the permutation distribution in all evaluations. This finding confirms that the performance of the original models is not achieved by chance, reinforcing their reliability.

**Figure 3:**
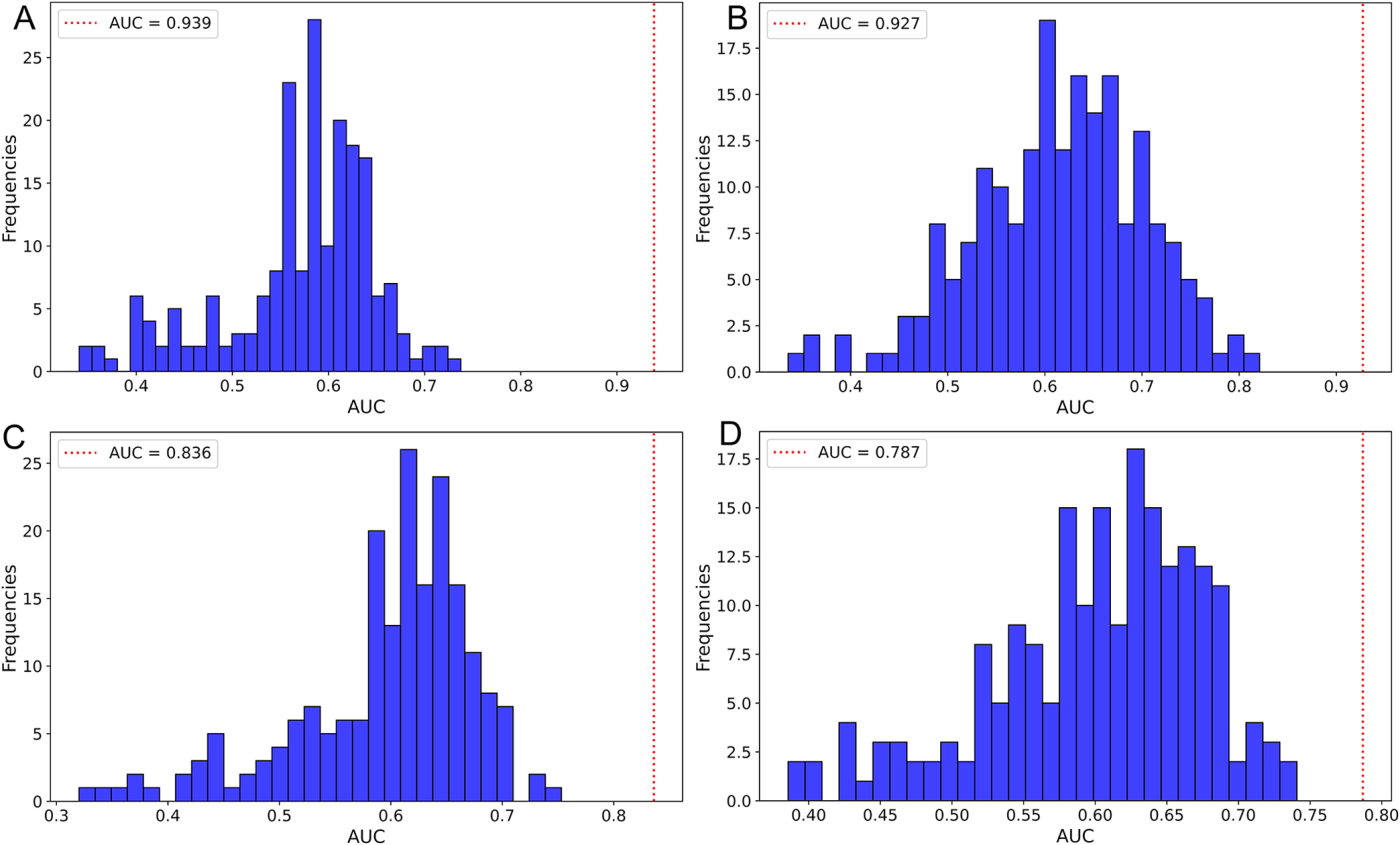
Permutation of the models for the training samples. Logistic Regression (dry data set) (A). PLS-DA (dry data set) (B). Random Forest (wet data set) (C). XGBoost (wet data set) (D). Red line = Real model; Blue Bars = permuted models.

## Discussion

This study presents a proof-of-concept analysis of a novel diagnostic approach for Chagas disease based on vibrational spectroscopy and machine learning. The development of rapid, accurate, and cost-effective diagnostic methods is essential for mitigating the global burden of CD, particularly in endemic regions where access to conventional diagnostic tests remains limited. Early detection is crucial for improving patient outcomes by enabling timely treatment and reducing disease progression.^17–21^ Implementing innovative diagnostic tools such as ATR-FTIR spectroscopy can strengthen public health efforts by facilitating large-scale screening programs and integrating new methodologies into clinical decision-making.

Two analytical protocols were evaluated using distinct data sets: dry and wet samples. Although dry analysis is conventionally preferred to minimize water interference, wet analysis offers the advantage of speed, requiring less than five minutes to process a sample, including spectral acquisition and preprocessing. This rapid turnaround is particularly relevant for point-of-care applications, where efficiency is critical in clinical decision-making. Spectral differences between *T. cruzi*-positive and negative samples were detected across the entire spectral range in both data sets, with the most pronounced variations occurring in the 1700–1500 cm^–1^ region, corresponding to peptide vibrations of proteins. Since preprocessing eliminates noise and interference signals,^22^ these spectral differences are likely attributable to biochemical alterations associated with the disease. The 1700–1500 cm^–1^ region between Chagas disease-positive and negative patients, particularly in Amide I (1700–1600 cm^–1^) and Amide II (1600–1500 cm^–1^) bands, reflect critical biochemical alterations linked to the pathophysiology of *T. cruzi* infection. In general, the Amide I band primarily corresponds to the C=O stretching vibration of the peptide bond, while the Amide II band is associated with N–H bending and C–N stretching modes. These vibrations provide critical information about the secondary structure of proteins, such as α-helices, β-sheets, and random coils.^23^ These bands, primarily associated with protein secondary structures and peptide bond vibrations, suggest changes in protein composition, structure, or post-translational modifications in the serum of infected individuals. The spectral differences observed between 1700–1500 cm^–1^ in Chagas patients are likely due to a combination of immune-driven protein overexpression, parasite-mediated proteolysis, oxidative protein damage, and tissue-derived biomarkers. These findings are consistent with the known pathophysiology of Chagas disease.^24–26^ A more detailed band assignment can be performed based on existing literature.^15,27^

Previous studies have demonstrated the potential of infrared spectroscopy in differentiating CD vectors and detecting the *T. cruzi*. Depickère et al.^28^ successfully utilized visible and near-infrared spectroscopy (VIS-NIR) to classify *Triatoma infestans*, *Triatoma sordida*, and *Triatoma guasayana*, achieving an overall accuracy of 97.2% in distinguishing nymphal instars and adult triatomines. Similarly, Tátila-Ferreira et al.^29^ employed NIR spectroscopy to detect the *T. cruzi* in *T. infestans* excreta, achieving 100% accuracy. However, this is the first study to evaluate the potential of infrared spectroscopy as a diagnostic tool for CD in humans.

Performance metrics demonstrated that the dry data set exhibited superior classification accuracy, with logistic regression achieving 93% sensitivity, 93% specificity, an F1-score of 93%, and an AUC of 0.99. The wet data set also demonstrated strong discriminatory ability, with XGBoost achieving 87% sensitivity, 87% specificity, an F1-score of 87%, and an AUC of 0.92. These results indicate that preprocessing methods effectively reduce water interference while preserving disease-related spectral signatures. Additionally, the wet protocol offers a practical advantage in clinical settings by enabling rapid testing in under five minutes, whereas the dry protocol requires approximately one hour per sample.

Following performance evaluation, quality validation is a critical step in predictive modeling analysis to mitigate bias, particularly when working with limited sample sizes.^30^ The permutation test performed on all original models using the training set data demonstrated a clear distinction between the frequency distributions of AUC values for the permuted and original models. This finding strongly indicates that the observed classification performance is not the result of random chance, reinforcing the reliability of the ML-based approach.

A comparison with established serological diagnostic methods further underscores the potential of ATR-FTIR spectroscopy. Commercial serological assays, such as ELISA, IIF, IHA, CLIA and rapid diagnostic tests (RDTs), exhibit variable sensitivity and specificity depending on antigen selection, population characteristics, and laboratory conditions.^11^ Our study demonstrated a sensitivity of 93% for the dry ATR-FTIR data set using logistic regression and 87% for the wet data set using XGBoost. In comparison, the highest sensitivity values reported in a recent multicenter study^31^ were observed for some EIA-based assays, with sensitivities reaching 100% in select cases, such as Biolisa Chagas Recombinante (Quibasa Química Básica, Brazil), and Anti Chagas SYM (Symbiosis Diagnostica, Brazil). Additionally, certain IIF and RDT platforms, such as Chagas IFA IgG + IgM (Vircell SL, Spain) and TR Chagas Bio-Manguinhos (Bio-Manguinhos, Fiocruz, Brazil), also exhibited 100% sensitivity. However, other serological tests showed considerable variability, with IHA kits demonstrating sensitivity values ranging from 71.2% to 93.7%, and some EIAs exhibiting sensitivity as low as 92.9%. These results suggest that ATR-FTIR spectroscopy, particularly with dry sample processing, achieves sensitivity comparable to some of the best-performing serological assays. While it does not surpass the highest sensitivity values of certain EIAs and IIF tests, it remains a highly effective screening tool.

Specificity is a critical parameter for ensuring that a diagnostic test minimizes false positives, particularly in low-prevalence settings. Our ATR-FTIR approach achieved 93% specificity in the dry data set and 87% specificity in the wet data set. In contrast, the specificity values of commercial serological tests varied widely. While some EIA kits, such as *T. cruzi* Ab—ELISA (Diapro Diagnostic Bioprobes SRL, Italy), reached 90.5% specificity, others, such as Chagatest ELISA lisado (Wiener Lab, Argentina), exhibited considerably lower specificity (78.2%).^31^ The IHA platforms showed even greater variability, with Chagas-HAI (Gold Analisa Diagnóstica, Brazil) achieving 94.9% specificity, while Chagatest HAI screening A-V (Wiener Laboratórios, Argentina) had a much lower specificity of 70.9%. Among IIF assays, specificity ranged from 62.1% to 88.6%, with Chagas IFA IgG + IgM exhibiting the lowest specificity, likely due to cross-reactivity with other parasitic diseases. These findings indicate that ATR-FTIR spectroscopy offers specificity comparable to or exceeding many conventional assays, particularly those employing crude antigens prone to cross-reactivity.

Diagnostic accuracy reflects the combined effects of sensitivity and specificity. In our study, ATR-FTIR spectroscopy with logistic regression for dry samples achieved an overall accuracy of 93%, whereas XGBoost for wet samples reached 87%. By comparison, among commercial assays, the highest accuracy values were reported for *T. cruzi* Ab—ELISA (95.5%) and Anti Chagas SYM (95.2%), as well as the RDT WL Check Chagas test (95.3%; Wiener Lab, Argentina).^31^ However, several serological assays exhibited lower overall accuracy due to imbalanced sensitivity and specificity. The Chagas IFA IgG + IgM test, despite reaching 100% sensitivity, showed significantly lower specificity, reducing its overall diagnostic accuracy. Similarly, IHA platforms demonstrated widely variable accuracy levels due to their limited specificity. The performance metrics obtained in this study suggest that vibrational spectroscopy, coupled with machine learning, could provide a reliable alternative for CD detection, particularly in resource-limited settings. However, further validation with larger data sets is necessary to confirm these findings and assess the technique’s reproducibility across diverse populations.

Despite these promising results, certain limitations must be acknowledged. This proof-of-concept analysis is based on a relatively small sample size (*n* = 100). However, this sample size was sufficient to demonstrate that ATR-FTIR spectroscopy can differentiate *T. cruzi*-positive samples from negative controls. Additional samples are currently being incorporated into the study to enhance the algorithm robustness and improve diagnostic accuracy.^32^ Future analyses will also include control groups with diseases that share serological or clinical overlap with CD, such as leishmaniasis, systemic lupus erythematosus, rheumatoid arthritis, and other relevant conditions. The inclusion of these differential diagnoses will be essential for assessing the model’s specificity in real-world clinical applications, ensuring its reliability as a screening tool.

## Conclusions

This study demonstrates the potential of vibrational spectroscopy combined with machine learning as a diagnostic tool for CD. Both analytical protocols tested—the dry data set (F1-score: 93%, AUC: 0.99) and the wet data set (F1-score: 87%, AUC: 0.92)—exhibited high discriminatory power in differentiating *T. cruzi*-positive from negative samples. Quality validation using permutation tests confirmed that the observed classification performance was not the result of random chance. Additionally, the wet protocol offers a significant advantage in terms of speed, enabling disease testing in under five minutes, making it a promising tool for point-of-care diagnostics. As this is an interim analysis, a larger data set is being integrated to refine and further validate the model. Future studies will focus on expanding the sample size and incorporating control groups with diseases that share clinical or serological similarities with CD to assess specificity and real-world applicability. If validated, this methodology could serve as a valuable tool in the early detection and management of CD, contributing to improved patient outcomes and more effective disease control strategies.

## Materials and Methods

### Ethics

This study was conducted following the principles outlined in the Declaration of Helsinki and received approval from the Institutional Review Board (IRB) of the Gonçalo Moniz Institute, Oswaldo Cruz Foundation (IGM-FIOCRUZ) in Salvador, Bahia, Brazil (protocol no. 67809417.0.0000.0040). Written informed consent was obtained from all participants prior to sample collection.

### Blood Samples

Anonymized human serum samples were obtained from the Central Public Health Laboratory of the State of Bahia (LACEN/BA) and the Hematology and Hemotherapy Foundation of the State of Bahia (HEMOBA Foundation). A total of 100 serum samples were included in this study. Of these, 49 were identified by LACEN/BA as positive for *T. cruzi* IgG antibodies. Serological confirmation was performed using two independent immunoassays: an enzyme immunoassay (Anti-Chagas SYM, Vyttra Diagnósticos, Leme, Brazil) and an electrochemiluminescence immunoassay (Chagas Elecsys, Roche Diagnostics, Mannheim, Germany), following the manufacturers’ protocols. The remaining 51 samples were obtained from volunteer blood donors at the HEMOBA Foundation. These samples tested negative for *T. cruzi* infection using commercial diagnostic tests and were also screened for syphilis, HIV-1/2, HTLV-1/2, hepatitis B, and hepatitis C, with all results being negative.

### ATR-FTIR Analysis

Serum samples were analyzed using an ATR-FTIR spectrometer (ALPHA II, Bruker), a reagent-free and rapid technique for biomolecular characterization.^33^ ATR-FTIR spectroscopy captures the interaction between infrared light and molecular structures, generating unique spectral signatures that reflect the biochemical composition of biofluids. This technique is considered “multiomic” due to its ability to detect diverse molecular components.^16^ Machine learning algorithms were employed to analyze the spectral data, identifying disease-specific patterns for classification. Further methodological details can be found in previous studies.^15,34,35^

Water in biofluid samples can introduce spectral interference, potentially masking disease-related signatures.^36^ To mitigate this effect, two processing protocols were employed. In dry analysis, 3 μL of serum was deposited onto the ATR-FTIR crystal, air-dried for 20 min, and subsequently analyzed. In the wet analysis, 3 μL of serum was placed directly onto the crystal, and spectra were acquired immediately. Each sample was analyzed in triplicate, with 32 coadded scans at a resolution of 4 cm^–1^ over the spectral range of 4000–400 cm^–1^. Spectral acquisition for each replicate required approximately 40 s.

### Machine Learning

A total of 300 spectra, obtained through triplicate measurements, were generated and subjected to ML-based classification. The spectral data matrix comprised approximately 1700 variables per spectrum, necessitating preprocessing and machine-leaning classification to extract meaningful patterns.

Spectral data preprocessing included truncation to the fingerprint region (1800–900 cm^–1^), which contains the most biologically relevant information. Baseline correction was performed using adaptive iteratively reweighted penalized least-squares (airPLS).^37^ To enhance spectral resolution and deconvolve overlapping bands, second derivative transformation was applied, with a window size of 9 for dry spectra and 21 for wet spectra.^38^

Data sets were randomly divided into training (70%) and test (30%) subsets. The training set was used to develop classification models, while test set, consisting of unseen spectra, was employed for model validation.^39^ Performance metrics, including sensitivity, specificity, accuracy, and area under the ROC curve (AUC), were assessed to evaluate model effectiveness.

Four ML classifiers were implemented. Logistic regression (LR) was applied to the dry data set, employing a statistical model that assigns probability scores and classifies samples based on a predefined threshold, typically 0.5.^40^ Partial least-squares discriminant analysis (PLS-DA) was also used for the dry data set, facilitating dimensionality while preserving variance and maximizing class separation.^41^ For the wet data set, two ensemble learning techniques were applied. The random forest (RF) classifier constructs multiple decision trees in parallel, each trained on a randomly selected subset of features, with final predictions aggregated through majority voting.^42^ Extreme gradient boosting (XGBoost) was also employed, representing an advanced boosting algorithm that sequentially builds decision trees while iteratively correcting classification errors.^43^ Given the spectral interference introduced by water in wet samples, extensive preprocessing and the use of advanced ML models were required to optimize classification accuracy.

To ensure model robustness and minimize classification bias, permutation tests were performed on the training set.^30^ During permutation, the class vector containing the correct diagnosis for each patient (0 for negative and 1 for positive) was partially shuffled to generate models based on incorrect classifications. A total of 200 permuted models were generated by randomly shuffling 40–60% of the class labels. The AUC was calculated for both permuted and correctly classified models using 5-fold cross-validation (*k* = 5). The distribution of AUC values from the permuted models was plotted to confirm the original models significantly outperformed chance-based classification, thereby validating reliability of the ML approach.
